# Death of Dr. Kirwin, of Ottawa

**Published:** 1859-06

**Authors:** 


### MISCELLANEOUS MEDICAL INTELLIGENCE.

### DEATH OF DR. KIRWIN, OF OTTAWA.

OTTAWA, ILL., May 6th, 1859.

At a special meeting of the "Ottawa City Medical Society," a resolution was passed, appointing a committee of three, consisting of Drs. Haid, Stout and Haughey, to draft resolutions expressive of the sense of the society, on the occasion of the death of Philip Kirwin, M.D.

In compliance with the above, the following preamble and resolutions were presented and unanimously adopted:

WHEREAS, In the disposition of Divine Providence, one of our number, Philip Kirwin, M.D., has been removed from this life,

*Resolved*, That we deeply regret the loss which we are called upon to sustain, in the death of one who, for many years, has been an active member of our society, and who, during that time, has endeared himself to its members by his estimable qualities as a gentleman, a citizen, and a physician.

*Resolved,* That we tender our heartfelt sympathies to his family and relatives, in their bereavement.

*Resolved,* That a copy of the foregoing preamble and resolutions be presented to the family of the deceased, and also to the journals of this city, and to the Chicago Medical Journal.

